# 

**Published:** 1854-04

**Authors:** 


### RECEIPTS

### FOR THE JOURNAL DURING THE MONTH OF APRIL.

### Illinois.

| | |
|---|---|
| Dr. Homer Cochron, | $2 00 |
| " George W. Brooks, | 2 00 |
| " Thos A Brown, | 2 00 |
| " N S Anderson, | 2 00 |
| " C. M Robinson, | 2 00 |
| " S. O. Long, | 2 00 |
| " Henry Bloxam, | 2 00 |
| " James M'Coy, | 2 00 |
| " S. A. Winslow, | 6 00 |
| " John Walker, | 2 00 |
| " D. H. Vosburg, | 2 00 |

### Iowa.

| | |
|---|---|
| Dr. J. H Graham, | $2 00 |
| " J. M Robertson, | 2 00 |
| " Wm Chamberlain, | 4 00 |
| " E. Vanesden, | 2 00 |
| " C. W. Butler, | 2 00 |

### Indiana.

| | |
|---|---|
| Dr. Simpson, | $5 00 |
| " Wm. H. Martin, | 3 00 |
| " Rufus Brown, | 2 00 |
| " W. Townsend, | 2 00 |
| " M. H. Bonnell, | 2 00 |
| " Rea & McConnell, | 2 00 |
| " Lathy & Jackson, | 6 00 |
| " William Wickham | 5 00 |
| " Miller, | 5 00 |

### Michigan.

| | |
|---|---|
| Dr. A. Platt, | $4 00 |
| " B. Hudson, | 2 00 |
| " A. D. Sargent, | 5 00 |
| " J. P. Hall, | 2 00 |

### Wisconsin.

| | |
|---|---|
| Dr. J. H Turner, | $2 00 |
| " C. W. Brigham, | 2 00 |

